# Polycystic Ovary Syndrome and the Risk of Premenstrual Disorders: A Nationwide Register-Based Study in Sweden

**DOI:** 10.1155/da/8226088

**Published:** 2025-06-25

**Authors:** Marion Opatowski, Jenny Deng, Qian Yang, Anna Sara Oberg, Carolyn E. Cesta, Donghao Lu

**Affiliations:** ^1^Institute of Environmental Medicine, Karolinska Institutet, Stockholm, Sweden; ^2^Division of Clinical Epidemiology, Department of Medicine Solna, Karolinska Institutet, Stockholm, Sweden; ^3^Department of Epidemiology, T.H. Chan School of Public Health, Boston, Massachusetts, USA; ^4^Department of Medical Epidemiology and Biostatistics, Karolinska Institutet, Stockholm, Sweden; ^5^Centre for Pharmacoepidemiology, Department of Medicine Solna, Karolinska Institutet, Stockholm, Sweden

**Keywords:** endocrine system, obesity, polycystic ovary syndrome, premenstrual disorders, premenstrual dysphoric disorder, psychiatric disorders, steroid hormones

## Abstract

**Background:** Polycystic ovary syndrome (PCOS) is one of the most common endocrine disorders affecting women of reproductive age. It has been linked to a range of psychiatric disorders. Although premenstrual disorders (PMDs) are characterized by psychiatric symptoms in tandem with hormone changes controlled by the endocrine system, the association between PCOS and PMDs remains unknown.

**Methods:** We conducted a nationwide register-based cohort study including 2,965,178 females during 2001–2018 in Sweden. Individuals with PCOS were identified from clinical diagnoses recorded in the Swedish national registers (*n* = 41,515) and PMDs were identified based on clinical diagnoses and prescriptions with a clear indication of PMDs during follow-up. Using multivariable Cox regression, hazard ratio (HRs) of PMDs were estimated by comparing individuals with PCOS to those without. To account for confounders such as genetics or family environment, we conducted sibling comparison (*N* = 160,566).

**Results:** During a median follow-up of 15.3 years, 1308 (1.9%) individuals with PCOS had a premenstrual disorder (PMD) (4.67/1000 person-years). Compared to individuals without PCOS they had more than doubled risk of PMDs (age-adjusted HR: 2.26, 95% CI 2.14– 2.39). The association was attenuated after adjustment for demographic and socioeconomic factors as well as for comorbid psychiatric disorders and obesity yet remained significant (HR: 1.54, 95% CI 1.46–1.63). The sibling comparison showed similar findings (full-adjusted HR: 1.61, 95% CI 1.36–1.92). The association between PCOS and PMDs remained statistically significant regardless of the presence of psychiatric comorbidities, with HR of 1.33 (95% CI 1.20–1.47) for individuals with psychiatric comorbidities and 1.55 (95% CI 1.45–1.65) for those without.

**Conclusions:** Our findings suggest that individuals diagnosed with PCOS were at increased risk for PMDs. This association could not be entirely explained by shared risk factors, including such that sisters share.

## 1. Introduction

Polycystic ovary syndrome (PCOS) is one of the most common endocrine disorders affecting women of reproductive age [1], with a prevalence varying between 5% and 20% worldwide [2, 3]. PCOS is characterized by hyperandrogenism, ovulatory disfunction, and polycystic ovarian morphology [1]. Although the etiology of PCOS remains elusive, some risk factors have been identified, such as a family history of PCOS [2, 4], and environmental [5] and epigenetic factors [6]. PCOS has been associated with several comorbidities including obesity [7], hyperinsulinemia, type 2 diabetes, cardiovascular diseases [2, 8], and psychiatric disorders [9–12]. With an increased risk of 50% for comorbid psychiatric disorders [9], it has been well-documented that patients with PCOS are commonly comorbid with depression and anxiety symptoms [1, 9, 11, 13]. In addition to shared genetics [9, 14], it is unclear whether endocrine factors play a role in the link between PCOS and psychiatric conditions [15].

In tandem with hormonal changes controlled by the endocrine system, premenstrual disorders (PMDs) are featured by a variety of emotional and physical symptoms occurring several days prior to menstruation. With an estimated prevalence of up to 30% [16], PMDs include premenstrual syndrome and premenstrual dysphoric disorder; the latter is recognized as a psychiatric disorder and affects 1%–8% of women of reproductive age [16–18], PCOS and PMDs share some characteristics. First, the underlying mechanisms of both disorders involve steroid hormones [1, 16]. Second, they share some symptomology (e.g., acne [2, 19]), metabolic syndrome [20, 21], and risk factors (e.g., obesity [8, 22] and adverse childhood events [23, 24]). One study reported a prevalence of 30% of PMDs among women with PCOS, compared to 15% among healthy controls [25]. However, the limited sample size of this study and lack of adjustment for confounders did not allow any conclusions to be drawn.

PCOS and PMDs may share some mechanisms. In this study, we aimed to investigate the association between PCOS and PMDs by comparing PMDs diagnoses among individuals with and without a diagnosis of PCOS. Knowledge about such potential comorbidity could provide evidence for health professions and patients with PCOS to refine the current clinical management and to better understand the mechanisms underlying both diseases.

## 2. Material and Methods

### 2.1. Data Source

The study was based on data from the Swedish national registers, all linked using the individual unique identification number [26]. These registers primarily included: (1) The National Patient Register (NPR) registering discharge records from inpatient and outpatient [27]; (2) the National Prescribed Drug Register which collects prescribed drug dispensing information from all Swedish pharmacies from July 2005 onwards; (3) the National Cause of Death Register; (4) the Longitudinal Integrated Database for Health Insurance and Labor Market studies (LISA) which integrates sociodemographic information for all Swedish residents aged 16 years and older since 1990; and (5) the total population register, which includes the National Migration Register and the Swedish Population and Housing Census.

### 2.2. Study Population and Design

We conducted a nationwide register-based cohort study followed by a sibling comparison. Based on the total population register, we included all female residents of reproductive age from January 1^st^, 2001 to December 31^st^, 2018. The reproductive age ranged from 15 (where > 90% of girls in Stockholm have their menarche [28]) and 52 (mean age for menopause in Sweden [29]). The study population were followed from the age of 15, or January 1st, 2001, whichever came later until age 52, emigration, death, or December 31st, 2018, whichever came first.

To control for unmeasured confounding shared between family members (e.g., genetics and familial environmental factors), we also conducted a sibling comparison. As described elsewhere [30], we identified full sisters who share biological parents recorded in the multigeneration register.

### 2.3. Ascertainment of PCOS

As described elsewhere [9], clinical diagnoses of PCOS were identified from the NPR using the Swedish version of the International Classification of Diseases (ICD) codes (Table S1). The NPR comprises all hospitalization records since 1987 and >80% of hospital-based outpatient records from 2001 [31]. We only included PCOS diagnosed after 1990 when the National Institute of Health (NIH) diagnostic criteria for PCOS were established [2]. To ensure specificity, women who had a comorbid condition which may have similar symptoms with PCOS were not classified as PCOS, in accordance with the NIH and the Rotterdam criteria that PCOS is a diagnosis of exclusion [2]. These conditions included pituitary adenoma, disorders of the pituitary glands, Cushing's syndrome, adrenogenital disorders including congenital adrenal hyperplasia, other disorders of the adrenal glands, and Turner's syndrome (Table S1).

Exposure to PCOS was treated as time-varying. Individuals diagnosed with PCOS during follow-up contributed person-time to the unexposed group until the date of first recorded PCOS diagnosis; and contributed person-time to the exposed group thereafter.

### 2.4. Ascertainment of PMDs

As described elsewhere [30], clinical diagnoses of PMDs were obtained from the NPR using ICD codes (Table S1). To capture diagnoses made in primary care, we also identified specific prescriptions for PMDs recorded in the Prescribed Drug Register. Briefly, we retrieved all dispensed prescriptions of antidepressants and hormonal contraceptives using Anatomical Therapeutic Chemical (ATC) codes; and then considered premenstrual disorder (PMD) case if any specific indication for PMDs was stated on the prescription (Table S1). Such indications included explicit mentioning of PMD diagnosis or instructing use of medication prior to menses.

### 2.5. Covariates

Year of birth, country of birth, and region of residence were obtained from the total population register. Educational level, disposable income, and cohabitation status were obtained from LISA. It is known that overweight/obesity and psychiatric disorders are positively associated with both PCOS and PMDs [7, 10, 22, 32]. To evaluate their influence, we identified clinical diagnoses of obesity and psychiatric disorders registered in the NPR using ICD codes for primary and secondary diagnoses before and at follow-up (Table S1). Obesity and psychiatric disorders were considered as time-varying covariates, while the other covariates were assessed at baseline.

### 2.6. Statistical Analyses

First, we accumulated exposed (PCOS) and unexposed person-times separately and compared the distribution of covariates between two groups. We calculated crude incidence rates (IRs) of PMDs in both groups.

Hazard ratio (HR) of PMDs and 95% CI were then estimated by comparing individuals with PCOS to those without, using Cox regression. The proportional hazards assumption was not violated according to the Schoenfeld residual test. We developed three models for adjustment. Model 1 was controlled for attained age as the underlying timescale. Model 2 was additionally adjusted for demographics (year of birth, country of birth, and region of residence) and socioeconomic status (educational level, income, and cohabitation status). Model 3 was further adjusted for psychiatric disorders and obesity.

Both psychiatric disorders and obesity are common among patients with PCOS [7, 9], and are associated with higher risk of PMDs [22, 32]. To test potential risk modification, we performed stratified analyses by preexisting psychiatric disorders and obesity separately.

To minimize the misclassification of PMDs from using indications on prescriptions, we conducted a sensitivity analysis limited to PMDs with a clinical diagnosis recorded in the NPR. Although prospective symptom charting is recommended by local clinical guidelines, we lacked information on the diagnostic process. To reduce potential false diagnoses, we further limited the analysis to those with two clinical diagnoses recorded in NPR at least 28 days apart.

Moreover, to assess the impact of possible delay in diagnosing PCOS, we excluded the unexposed time preceding the PCOS diagnosis from the regression models for individuals with PCOS

Finally, to account for changes in diagnostic criteria and increased awareness of PCOS and PMDs over time, we conducted a sensitivity analysis by stratifying the regression by calendar year at follow-up. For this, we split the follow-up using 5-year intervals.

All analyses were conducted for the general population cohort and for the sibling cohort. For sibling analyses, we used Cox proportional hazard regression stratified on sibling set to compare PMDs rates among siblings discordant for PCOS, with attained age as the underlying timescale.

This study has been approved by the Swedish Ethical Review Authority (2013/1849–31/2, 2020–03812). Data has been collected by authorities for administrative purposes; a written consent form is therefore waived for register-based studies.

Data were prepared in SAS 9.4 (SAS Institute Inc.) and analyzed using Stata 17.0 (STATA, College Station, TX). *p*-Value of less than 0.05 was considered statistically significant.

## 3. Results

### 3.1. Population Selection

A total of 2,935,728 individuals were included in the study, among which 1.4% (*n* = 41,508) were diagnosed with PCOS before or during the follow-up. A total of 53,683 siblings discordant for PCOS were identified in the study population. Among them, 31.3% (*n* = 16,785) were diagnosed with PCOS before or during the follow-up.

### 3.2. Individual Characteristics

Individuals contributing person-time to the PCOS group were more likely to be older, born in Sweden, and single compared to the individuals contributing person-time to the PCOS-free group (Table 1). Although they had higher education attainment, they had lower income. Moreover, they were more likely to have a clinical diagnosis of psychiatric disorders or obesity.

Similar trends were noted among siblings, except for the country of birth, with over 87% of individuals contributing person-time to both PCOS and non-PCOS groups born in Sweden.

### 3.3. Risk of PMDs

During a median follow-up time of 15.3 years, 68,548 individuals received a diagnosis of PMDs and 1,308 (1.9%) of them were subsequent to a PCOS diagnosis (Table 2). The incidence rate of PMDs was 4.67 (95% CI 4.42–4.93) per 1,000 person-years in those with PCOS and 1.77 (95% CI 1.76–1.78) in those without PCOS. Similar IRs were observed in the siblings cohort.

Compared to individuals without PCOS, individuals with PCOS had more than doubled risk of PMDs (HR: 2.26, 95% CI 2.14–2.39; Table 2, Model 1) when accounting for age at follow-up. When adjusting for demographics, the association between PCOS and risk of PMDs was attenuated yet remained significant (HR: 1.59, 95% CI 1.50–1.68). With additional adjustment for psychiatric disorders and obesity diagnoses, PCOS was associated with a 48% higher risk of PMDs (HR: 1.48, 95% CI 1.40–1.56). Among siblings, individuals with PCOS were still at higher risk for PMDs than individuals free from PCOS, after adjustment for age, demographics, psychiatric disorders and obesity (HR: 1.61, 95% CI 1.36–1.92).

When stratifying by psychiatric disorders, the increased risk of PMDs among women with PCOS appeared to be higher among individuals without any psychiatric comorbidity (HR: 1.55, 95% CI 1.45–1.65; Table 3), *p*-for-interaction = 0.014, Table 3), although it remained elevated also among those with psychiatric comorbidity (HR: 1.33, 95% CI 1.20–1.47). We observed no difference in the risk estimates when stratifying by obesity (*p*-for-interaction = 0.298). Similar patterns were noted for the sibling comparison.

### 3.4. Sensitivity Analyses

When restricting PMD diagnoses to at least one clinical diagnosis or at least two clinical diagnoses ≥28 days apart, the risk of PMDs associated with PCOS remained higher. Clinical diagnosis of PMDs was associated with a 97% elevated risk of PMDs (adjusted HR: 1.97, [95% CI 1.84–2.11], Table S2). Consistent estimates were observed in the sibling comparison. Similarly, women with at least two clinical diagnoses ≥28 days apart had higher risk of experiencing PMDs (adjusted HR: 2.16, [95% CI 1.91–2.44], Table S2). The association was smaller for the sibling comparison, but still statistically significant and consistent with the main analysis.

Excluding the unexposed time prior to PCOS diagnosis yielded results similar to those of the main analyses. The adjusted HR was 1.48 (95% CI: 1.40–1.57, Table S3, Model 3) in the population analysis and 1.67 (95% CI: 1.40–1.99) in the sibling comparison. When stratifying by calendar year, the association between PCOS and PMDs remained statistically significant. Before 2006, the HR was higher than after (adjusted HR: 3.50 (2.68–4.58 versus 1.66 [1.42–1.93] for 2006–2010, Table S4). Similar trends were observed in the sibling comparison.

## 4. Discussion

### 4.1. Principal Findings

In this nationwide cohort study of almost 3 million individuals followed for a median time of 15 years, we found that individuals diagnosed with PCOS had a higher risk of PMDs. Importantly, the association was not fully explained by several known risk factors or predictors shared between PCOS and PMDs, such as obesity, psychiatric disorders, as well as genetic and familial environment shared by full sisters.

### 4.2. Results in the Context of What Is Known

Previous studies have reported correlations between PCOS and several psychiatric disorders [9, 33, 34]. A systematic review concluded positive associations between PCOS and depression, anxiety, bipolar disorder, and obsessive–compulsive disorder [34]. Our data further suggests that PCOS is positively associated with PMDs, with an increased risk of more than 50% also after adjustment for several shared risk factors. Such association was largely comparable to the relationships between PCOS and psychiatric disorders estimated in other register-based studies and reviews [9, 33, 34]. Notably, there is one relatively small study reporting a prevalence of 30% of PMDs among patients with PCOS, while the prevalence reached 15% among healthy individuals [25]. However, our study is the first to formally assess the risk for PMDs among individuals with PCOS.

### 4.3. Clinical and Research Implications

Several mechanisms could explain the association between PCOS and PMDs. It has been suggested that the common clinical features of PCOS (infertility, hirsutism, acne, male-patterned hair loss, and obesity) contribute to depression and anxiety symptoms [9–11]. However, even after treatment for hirsutism and improvement for symptoms, patients with PCOS did not report significant reduction of their mental symptoms [35]. In several studies, depression or anxiety symptoms remained elevated even after matching or controlling for infertility, BMI, or family history of depression [10, 36, 37]. This suggests that the clinical features of PCOS may not fully explain the association between PCOS and psychiatric disorders. Still, the mechanisms involved in the link between PCOS and PMDs could differ from those leading to the association with other psychiatric disorders. This is supported by the stratified analyses in this study, where the risk of PMDs was higher among women regardless of the presence of psychiatric disorder.

It is plausible that neuroactive steroids account for the observed link between PCOS and PMDs. Allopregnanolone (ALLO) is a metabolite of progesterone and a modulator of the gamma-aminobutyric acid (GABA) receptor, which is a pivotal regulator of stress, anxiety, or vigilance [15]. Although the etiology of PMDs remains unclear, recent research has highlighted altered sensitivity to reproductive hormones modulations and a key role of GABA receptor plasticity and sensitivity to ALLO [16, 38, 39]. Women with PCOS tend to have higher levels of progesterone and ALLO, but they also may present a decreased sensitivity of GABA receptors to ALLO sedative effects [40].

PCOS and PMDs may also share some genetic factors. For example, genetic variants of catechol-*o*-methyl transferase (COMT) [41, 42], monoamine oxidase (MAO) [41, 42], brain derived neurotrophic factor (BDNF) [41, 43], or estrogen receptors (ESR1 and ESR2) [41, 44], have been associated with both diseases. If genetic factors were responsible for the observed association, we would expect an attenuated association when comparing within full sisters, who share about half of the genetic makeup. However, the sibling comparison yielded a similar or even stronger association, indicating that shared genetic factors may not explain our finding.

### 4.4. Strengths and Limitations

This study is based on a nationwide register-based cohort study of about 2.9 million individuals. The large sample size improves the precision of statistical estimates. The prospectively collected data and complete follow-up limit the risk of common biases in observational studies, such as recall bias and selection bias. A sibling comparison enables further adjustment for unmeasured factors that are shared by family members.

However, this study has several limitations. First, some potential confounders were not available from the registers, such as body mass index [7, 22]. We used ICD-10 codes for obesity or overweight as a proxy for high body mass index. Of note, obesity and overweight identified in the registers may represent the most severe cases, potentially leading us to misclass some overweight individuals as non-overweight. Still, if obesity/overweight is a confounder of the studied association, we would expect to observe an attenuated or null association among the women diagnosed with obesity/overweight. Our stratified analyses yielded similar associations between PCOS and PMDs women with and without obesity/overweight, respectively; particularly, the remained association among those with obesity suggests that obesity does not completely explain this link between PCOS and PMDs. Although we lacked information on timing of menarche [45, 46], smoking status [47, 48], and adverse child events [23, 24], which are potential confounders for the studied association, sibling comparison may have accounted for these factors to some extent [49, 50].

Second, misclassifications may have occurred for PCOS and PMDs diagnoses. Individuals with PCOS may have been classified into the reference group. Indeed, we were not able to identify diagnoses made in primary care, as well as individuals with PCOS not yet seeking healthcare service. Also, because it can take years to receive a proper diagnosis of PCOS, women may have been wrongly classified in the reference group before diagnosis [51]. Moreover, patients with PCOS diagnosed by specialists or treated medically might represent the most severe PCOS. Similar misclassification applies to PMDs as well [16, 52]. In addition, PMDs diagnosis is complex and require symptom assessment over two distinct menstrual cycles [16], which cannot be verified in the registers. Some individuals may then have been wrongly diagnosed with PMDs. Together, these misclassifications would likely contribute to an attenuation of a potential association. Sensitivity analyses that restricted the PMDs to clinical diagnoses or at least two diagnoses yielded similar results, indicating a limited effect of this potential bias. In our study, the mean age of diagnosis was 27.7 (SD 6.2) years for PCOS, and 35.4 (SD 7.9) years for PMDs, respectively. This is in line with other reports on the age of first diagnosis of these conditions [52–54].

Moreover, the introduction of the Rotterdam criteria for PCOS diagnosis in 2003 may have influenced the identification of PCOS, leading to an increase in the number of women diagnosed after 2003 [9, 51]. To account for this, as well as the growing awareness of PCOS and PMDs over time, we stratified the analysis by year of follow-up. The association remained statistically significant over time, demonstrating the robustness of the relationship. The HR was notably higher before 2006, possibly reflecting that the earlier diagnoses predominantly captured the most severe cases of PCOS and PMDs.

It is worth noting that individuals with PCOS may have had increased interaction with healthcare services and a higher likelihood of undergoing screening for PMDs, potentially introducing a surveillance bias. However, the prevalence of PMDs diagnosis after PCOS in our study (3.1%) falls within the lower range of the prevalence reported in the literature [16, 18].

Last, some PCOS patients may not experience any menstruation as part of the syndrome and were therefore not at risk for PMDs during that period. Many women with PCOS who receive treatment can regain normal menstruation. Notably, oral contraceptives are common treatments for both PCOS [33] and PMDs [16]. By treating PMDs symptoms, use of contraceptives could also reduce likelihood that women with PCOS are diagnosed with PMDs. As such, a potential association of PCOS with PMD would be mitigated and less pronounced than it would be in the absence of such treatment. Last, information on PCOS phenotypes or females' androgen status is not available from national registers. Further studies using PCOS status and distinguishing between premenstrual syndrome versus premenstrual dysphoric disorders would be valuable.

## 5. Conclusions

This large nationwide cohort study suggests that individuals diagnosed with PCOS faced a higher risk of PMDs. It is the first study, to our knowledge, to estimate this association, and it sheds valuable light into the potential mechanisms behind PCOS and PMDs. It is recently recognized that PMDs entail a range of significant negative health outcomes through reproductive lifespan, such as perinatal depression, early menopause, suicidal behavior, and even premature death [30, 55–57]. Understanding their link with PCOS may help advance future research on common etiological pathways and inform more integrated approaches to women's mental and reproductive health.

## Figures and Tables

**Table 1:** Baseline characteristics of individuals with and without polycystic ovary syndrome, person-years (%).

Characteristics	Total population		Sibling population	
	No PCOS	PCOS	No PCOS	PCOS
Total	38,738,328	280,391	424,537	117,140
Age (in years)				
15–19	5,802,704 (15.0)	8416 (3.0)	101,615 (23.9)	3756 (3.2)
20–24	6,026,194 (15.6)	33,834 (12.1)	108,300 (25.5)	15,510 (13.2)
25–29	5,865,245 (15.1)	62,763 (22.4)	86,756 (20.4)	27,643 (23.6)
30–34	5,639,910 (14.6)	68,660 (24.5)	57,689 (28.6)	28,660 (24.5)
35–39	5,671,979 (14.6)	54,257 (19.4)	36,254 (8.5)	21,506 (18.4)
40–44	5,157,593 (13.3)	33,615 (12.0)	21,026 (5.0)	12,910 (11.0)
≥45	4,574,705 (11.8)	18,845 (6.7)	12,896 (3.0)	7155 (6.1)
Year of birth				
1960–1969	10,001,150 (25.8)	30,999 (11.1)	30,971 (7.3)	11,840 (10.1)
1970–1979	11,305,141 (29.2)	96,975 (34.6)	100,262 (23.6)	38,833 (33.1)
1980–1989	11,288,110 (29.1)	115,509 (41.2)	205,461 (48.4)	50,070 (42.7)
1990–2003	6,143,928 (15.9)	36,908 (13.2)	87,843 (20.7)	16,396 (14.0)
Calendar year at follow-up				
2001–2005	9,622,336 (24.8)	17,658 (6.3)	110,169 (26.0)	7376 (6.3)
2006–2010	11,064,829 (28.6)	54,222 (19.3)	131,032 (30.9)	22,808 (19.5)
2011–2015	11,534,140 (29.8)	108,946 (38.9)	123,795 (29.2)	45,683 (39.0)
2016–2018	6,517,025 (16.8)	99,565 (35.5)	59,541 (14.0)	41,273 (35.2)
Country of birth				
Sweden	28,286,836 (73.0)	214,681 (76.6)	372,653 (87.8)	104,371 (89.1)
Other	10,451,492 (27.0)	65,711 (23.4)	51,884 (12.2)	12,769 (10.9)
Region of residency				
North	5,932,690 (15.3)	49,537 (17.7)	66,726 (15.7)	22,086 (18.8)
Middle	22,489,808 (58.1)	158,924 (56.7)	258,907 (61.0)	64,179 (54.8)
South	8,430,136 (21.8)	70,747 (25.2)	97,626 (23.0)	30,798 (26.3)
Unknown	1,885,695 (4.9)	1183 (0.4)	1278 (0.3)	77 (0.0)
Educational level				
Primary	5,509,113 (14.2)	38,790 (13.8)	91,750 (21.6)	15,053 (12.8)
Secondary	17,817,850 (46.0)	154,180 (55.0)	195,455 (46.0)	67,088 (57.3)
Collage and beyond	6,567,823 (17.0)	74,725 (26.7)	62,339 (14.7)	32,076 (27.4)
Unknown	8,843,544 (22.8)	12,696 (4.5)	74,993 (17.7)	2923 (2.5)
Income				
<Q1	7,478,310 (19.3)	83,784 (29.9)	103,660 (24.4)	33,985 (29.0)
Q1–<Q2	7,629,409 (19.7)	72,658 (25.9)	80,920 (19.1)	29,019 (24.8)
Q2–<Q3	7,765,458 (20.1)	69,426 (24.8)	84,301 (19.9)	30,801 (26.3)
≥Q3	7,797,815 (20.1)	46,512 (16.6)	87,782 (20.7)	21,445(18.3)
Unknown	8,067,338 (20.8)	8013 (2.9)	67,874 (16.0)	1890 (1.6)
Cohabitation				
Yes	9,512,596 (24.6)	95,844 (34.2)	66,085 (15.6)	36,745 (31.4)
No	21,158,394 (54.6)	176,534 (63.0)	290,578 (68.4)	78,505 (67.0)
Unknown	8,067,338 (20.8)	8013 (2.8)	67,874 (16.0)	1890 (1.6)
Clinical diagnosis of:				
Psychiatric disorder				
Yes	2,770,667 (7.2)	53,000 (18.9)	36,263 (8.5)	22,162 (18.9)
No	35,967,660 (92.8)	227,391 (81.1)	388,273 (91.5)	94,978 (81.1)
Obesity				
Yes	442,790 (1.1)	31,275 (11.2)	8560 (2.0)	12,788 (10.9)
No	38,295,536 (98.9)	249,116 (88.8)	415,977 (98.0)	104,352 (89.1)

*Note*: Q1–Q3, interquartile ranges.

Abbreviations: PCOS, polycystic ovary syndrome; PYs, person-years.

**Table 2:** Association between polycystic ovary syndrome and subsequent risk of premenstrual disorders.

Study design	No PCOS *N* (IR)	PCOS *N* (IR)	Model 1 HR^a^ (95% CI)	Model 2 HR^b^ (95% CI)	Model 3 HR^c^ (95% CI)
All population	68,548 (1.77)	1308 (4.66)	2.26 (2.14–2.39)	1.59 (1.50–1.68)	1.48 (1.40–1.56)
Siblings	829 (1.95)	567 (4.84)	1.84 (1.56–2.16)	1.70 (1.44–2.01)	1.61 (1.36–1.92)

*Note: N*, number of premenstrual disorder cases

Abbreviations: CI, confidence interval; HR, hazard ratio; IR, incidence rate per 1000 person-years; PCOS, polycystic ovary syndrome.

^a^HR was adjusted for attained age as the underlying timescale.

^b^HR, was additionally adjusted for year of birth (1960–1969, 1970−1979, 1980–1989, or 1990–2003), calendar year at follow-up (2001–2005, 2006−2010, 2011–2015, or 2016–2018), country of birth (Sweden or other), region of residency (north, middle, south, or unknown), educational level (primary, secondary, collage and beyond, or unknown), income (by quartiles, or unknown), and cohabitation (yes, no, or unknown).

^c^HR, was additionally adjusted for clinically diagnosed psychiatric disorder (yes or no) and obesity (yes or no).

**Table 3:** Association between PCOS and subsequent risk of PMDs, stratified on clinically diagnosed psychiatric disorders and obesity.

Comparison groups	Population comparison		Sibling comparison	
	*N* (IR)	Model 3 HR^a^ (95% CI)	*N* (IR)	Model 3 HR^a^ (95% CI)
By psychiatric disorder				
No	—	—	—	—
No PCOS	99,565 (1.62)	1.00	682 (1.76)	1.00
PCOS	939 (4.13)	1.55 (1.45–1.65)	408 (4.30)	1.62 (1.33–1.97)
Yes	—	—	—	—
No PCOS	10,456 (3.77)	1.00	147 (4.05)	1.00
PCOS	369 (6.96)	1.33 (1.20–1.47)	159 (7.17)	1.60 (1.05–2.43)
*p*-Value for interaction	—	0.014	—	0.957
By obesity				
No	—	—	—	—
No PCOS	67,331 (1.76)	1.00	803 (1.93)	1.00
PCOS	1168 (4.69)	1.49 (1.41 1.58)	514 (4.93)	1.63 (1.37–1.95)
Yes	—	—	—	—
No PCOS	1217 (2.75)	1.00	26 (3.04)	1.00
PCOS	140 (4.48)	1.35 (1.14–1.61)	53 (4.14)	1.27 (0.52–3.10)
*p*-Value for interaction	—	0.298	—	0.592

*Note: N*, number of PMDs.

Abbreviations: CI, confidence interval; HR, hazard ratio; IR, incidence rate per 1000 person-years; PMDs, premenstrual disorders; PY, person-years.

^a^HR, attained age was used as the underlying timescale and models were adjusted for year of birth (1960–1969, 1970−1979, 1980–1989, or 1990–2003), calendar year at follow-up (2001–2005, 2006−2010, 2011–2015, or 2016–2018), country of birth (Sweden or other), region of residency (north, middle, south, or unknown), educational level (primary, secondary, collage and beyond, or unknown), income (by quartiles, or unknown), cohabitation (yes, no, or unknown), clinically diagnosed psychiatric disorder (yes or no, when stratified on obesity), and obesity (yes or no, when stratified on psychiatric disorders).

## Data Availability

Due to Swedish privacy protection governed by the General Data Protection Regulation, access to register data can only be granted after ethical approval by the ethics review authority. Information can be found at the Swedish National Board of Health and Welfare (https://bestalladata.socialstyrelsen.se/, email: registerservice@socialstyrelsen.se) and/or Statistics Sweden (https://www.scb.se/vara-tjanster/bestall-data-och-statistik/, email: scb@scb.se). Upon reasonable request codes for data analysis can be shared.

## Supporting Information

Supporting Information Supporting Information include Table S1 for diagnosis codes and Tables S2–S4 for sensitivity analyses.
