# Supplementary material for: Polycystic Ovary Syndrome and the Risk of Premenstrual Disorders: A Nationwide Register-Based Study in Sweden
Source: Depress Anxiety. 2025 Jun 25;2025:8226088. doi: 10.1155/da/8226088 (PMC12221545; doi:10.1155/da/8226088)
Supplement: Supporting Information — include Table S1 for diagnosis codes and Tables S2–S4 for sensitivity analyses. [file 8226088.f1.docx]

**Supplementary materials**

| **Disorder** | **ICD-8** | **ICD-9** | **ICD-10** | **ATC** |
| --- | --- | --- | --- | --- |
| Polycystic ovary syndrome |  |  |  |  |
| Polycystic ovary syndrome | - | 256E | E28.2 |  |
| Pituitary adenoma | 226.20 | 277D | D352 |  |
| Disorders of the pituitary glands | 253 | 253 | E22 |  |
| Disorders of the adrenal gland | 255 | 255 | E24; E25; E27 |  |
| Turner Syndrome | 759.5 | 758G | O96 |  |
| Premenstrual disorders |  |  |  |  |
| Premenstrual disorders |  | 625E | N943 |  |
| Antidepressant |  |  |  | N06AB, N06AX, N06AA |
| Oral contraceptives |  |  |  | G03A, G02B |
| Covariates |  |  |  |  |
| Obesity |  |  | E65; E66 |  |
| Psychiatric disorders | 290-319 | 290-319 | F10-F90 |  |

**Table S1. Codes for identification of** **polycystic ovary syndrome, premenstrual disorders and covariates**

Abbreviations: ICD, International Classification of Diseases; ATC, Anatomical Therapeutic Chemical codes

**Table S2. Sensitivity analyses for association between polycystic ovary syndrome and subsequent risk of premenstrual disorders.**

| **Comparison groups** | **N (IR)** | **Model 1**  **HR¹ (95% CI)** | **Model 2**  **HR² (95% CI)** | **Model 3**  **HR³ (95% CI)** |
| --- | --- | --- | --- | --- |
| **Restriction to PMDs recorded as clinical diagnoses in the Patient Register** | | | | |
| Population comparison |  |  |  |  |
| No PCOS | 36,096 (0.93) | 1.00 | 1.00 | 1.00 |
| PCOS | 878 (3.13) | 2.85 (2.67 – 3.05) | 2.14 (2.00 – 2.29) | 1.97 (1.84 – 2.11) |
| Sibling comparison |  |  |  |  |
| No PCOS | 490 (1.15) | 1.00 | 1.00 | 1.00 |
| PCOS | 367 (3.13) | 2.17 (1.76 – 2.69) | 2.07 (1.66 – 2.58) | 1.93 (1.54 – 2.41) |
| **Restriction to PMDs recorded at least twice in ≥28 days apart in the Patient Register** | | | | |
| Population comparison |  |  |  |  |
| No PCOS | 10,380 (0.27) | 1.00 | 1.00 | 1.00 |
| PCOS | 267 (0.95) | 2.95 (2.62 – 3.34) | 2.37 (2.09 – 2.67) | 2.16 (1.91 – 2.44) |
| Sibling comparison |  |  |  |  |
| No PCOS | 190 (0.45) | 1.00 | 1.00 | 1.00 |
| PCOS | 112 (0.96) | 1.77 (1.23 – 2.55) | 1.70 (1.16 – 2.50) | 1.69 (1.14 – 2.49) |

 Abbreviations: PCOS, polycystic ovary syndrome; N, number of PMDs; IR, incidence rate per 1000 person-years; HR, hazard ratio; CI, confidence interval; PMDs, premenstrual disorders.
¹ HR was adjusted for attained age as the underlying timescale.
² HR was additionally adjusted for year of birth (1960-1969, 1970-1979, 1980-1989, or 1990-2003), calendar year at follow-up (2001-2005, 2006-2010, 2011- 2015, or 2016-2018), country of birth (Sweden or other), region of residency (north, middle, south, or unknown), educational level (primary, secondary, collage and beyond, or unknown), income (by quartiles, or unknown), cohabitation (yes, no, or unknown).
³ HR was additionally adjusted for clinically diagnosed psychiatric disorder (yes or no) and obesity (yes or no).

**Table S3. Sensitivity analysis:** association between polycystic ovary syndrome and subsequent risk of premenstrual disorders: exclusion of time prior PCOS diagnosis.

|  | **No PCOS** | **PCOS**  N (IR) | **Model 1**  HR^1^ (95% CI) | **Model 2**  HR^2^ (95% CI) | **Model 3**  HR^3^ (95% CI) |
| --- | --- | --- | --- | --- | --- |
|  | N (IR) |  |  |  |  |
| All population | 68,063 (1.77) | 1,308 (4.66) | 2.26  (2.14 – 2.39) | 1.59  (1.50 – 1.68) | 1.48  (1.40 – 1.57) |
| Siblings | 619 (2,15) | 567 (4.84) | 1.85  (1.57 – 2.18) | 1.76  (1.48– 2.08) | 1.67  (1.40 – 1.99) |

Abbreviations: PCOS, polycystic ovary syndrome; N, number of premenstrual disorder cases; IR, incidence rate per 1000 person-years; HR, hazard ratio; CI, confidence interval.
¹ HR was adjusted for attained age as the underlying timescale.
² HR: was additionally adjusted for year of birth (1960-1969, 1970-1979, 1980-1989, or 1990-2003), calendar year at follow-up (2001-2005, 2006-2010, 2011- 2015, or 2016-2018), country of birth (Sweden or other), region of residency (north, middle, south, or unknown), educational level (primary, secondary, collage and beyond, or unknown), income (by quartiles, or unknown), cohabitation (yes, no, or unknown).
³ HR: was additionally adjusted for clinically diagnosed psychiatric disorder (yes or no) and obesity (yes or no).

**Table S4. Association between PCOS and subsequent risk of PMDs, stratified on calendar year at follow-up.**

| **Comparison groups** | **Population comparison** | | **Sibling comparison** | |
| --- | --- | --- | --- | --- |
|  | N (IR) | Model 3  HR¹ (95% CI) | N (IR) | Model 3  HR¹ (95% CI) |
| **By calendar year** |  |  |  |  |
| 2001-2005 |  |  |  |  |
| No PCOS | 6,437 (0.67) | 1.00 | 75 (0.68) | 1.00 |
| PCOS | 54 (3.06) | - 1. (2.68 – 4.58) | 22 (2.98) | 2.22 (1.06– 4.68) |
| 2006-2010 |  |  |  |  |
| No PCOS | 15,073 (1.36) | 1.00 | 170 (1.30) | 1.00 |
| PCOS | 163 (3.01) | 1.66 (1.42– 1.93) | 60 (2.63) | 1.52 (0.97 – 2.39) |
| 2011-2015 |  |  |  |  |
| No PCOS | 25,931 (2.25) | 1.00 | 324 (2.62) | 1.00 |
| PCOS | 528 (4.85) | 1.57 (1.44 – 1.71) | 240 (5.25) | 1.74 (1.32 – 2.28) |
| 2016-2018 |  |  |  |  |
| No PCOS | 21,207 (3.24) | 1.00 | 260 (4.37) | 1.00 |
| PCOS | 563 (5.65) | 1.30 (1.19 – 1.41) | 245 (5.94) | 1.39 (0.98 – 1.97) |
| p-value for interaction |  | <0.001 | IR, incidence rate | 0.647 |

 Abbreviations: PY, person-years; PMDs, premenstrual disorders; N, number of PMDs; IR, incidence rate per 1000 person-years; CI, confidence interval; HR, hazard ratio.
¹ HR: attained age was used as the underlying timescale and models were adjusted for year of birth (1960-1969, 1970-1979, 1980-1989, or 1990-2003), calendar year at follow-up (2001-2005, 2006-2010, 2011- 2015, or 2016-2018), country of birth (Sweden or other), region of residency (north, middle, south, or unknown), educational level (primary, secondary, collage and beyond, or unknown), income (by quartiles, or unknown), cohabitation (yes, no, or unknown), clinically diagnosed psychiatric disorder (yes or no, when stratified on obesity) and obesity (yes or no, when stratified on psychiatric disorders).
